# Phase 1 Study of the Effects of the Tuberculosis Treatment Pretomanid, Alone and in Combination With Moxifloxacin, on the QTc Interval in Healthy Volunteers

**DOI:** 10.1002/cpdd.898

**Published:** 2020-12-30

**Authors:** Mengchun Li, George A. Saviolakis, Wael El‐Amin, Mamodikoe K. Makhene, Blaire Osborn, Jerry Nedelman, Tian J. Yang, Daniel Everitt

**Affiliations:** ^1^ TB Alliance New York New York USA; ^2^ DynPort Vaccine Company (DVC), LLC, a GDIT company Frederick Maryland USA; ^3^ Division of Microbiology and Infectious Diseases, National Institute of Allergy and Infectious Diseases National Institutes of Health Bethesda Maryland USA; ^4^ Office of Clinical Pharmacology United States Food and Drug Administration Silver Spring Maryland USA

**Keywords:** pretomanid, PA‐824, moxifloxacin, thorough QT study, tuberculosis

## Abstract

Tuberculosis (TB) continues to be a serious threat to public health throughout the world. Newer treatments are needed that could offer simplified regimens with activity against both drug‐sensitive and drug‐resistant bacilli, while optimizing safety. Pretomanid (PA‐824), a nitroimidazooxazine compound, is a new drug for the treatment of pulmonary TB that was recently approved in the United States and Europe in the context of a regimen combined with bedaquiline and linezolid. This phase 1 double‐blind, randomized, placebo‐controlled crossover study specifically examined the effect of single‐dose administration of pretomanid 400 or 1000 mg and pretomanid 400 mg plus moxifloxacin 400 mg on the QTc interval in 74 healthy subjects. Subjects were fasting at the time of drug administration. Pretomanid concentrations following single 400‐ or 1000‐mg doses were not associated with any QT interval prolongation of clinical concern. Moxifloxacin did not alter the pharmacokinetics of pretomanid, and the effect of pretomanid 400 mg plus moxifloxacin 400 mg on the individually corrected QT interval was consistent with the effect of moxifloxacin alone. Both drugs were generally well tolerated. Although supratherapeutic exposure of pretomanid relative to the now‐recommended dosing with food was not achieved, these findings contribute to the favorable assessment of cardiac safety for pretomanid.

Tuberculosis (TB), typically caused by *Mycobacterium tuberculosis* (MTB) in humans, is an airborne disease that can be transmitted from person to person. Although transmission often leads to latent infection, which is neither transmissible nor associated with symptoms, approximately 5% to 10% of the estimated 1.7 billion people infected with MTB will develop active disease at some point in their lives.^1^, ^2^ Active TB was declared a global emergency by the World Health Organization (WHO) in 1993 and has continued to pose a serious threat to public health worldwide.^2^ According to the WHO, there were an estimated 10.0 million new cases of TB in 2019.^2^ Approximately 1.2 million TB‐related deaths occurred among people negative for human immunodeficiency virus (HIV) and an additional 208,000 deaths among those positive for HIV. Tuberculosis is 1 of the top 10 leading causes of death worldwide and the leading cause of death from a single infectious agent, placing it above HIV/acquired immune deficiency syndrome.^2^ The control of TB is a major challenge for health authorities, and newer treatments are needed that could simplify existing regimens, shorten treatment duration, be active against drug‐sensitive as well as drug‐resistant bacilli, and have a favorable safety profile when taken in combination with other medications for TB.^3^, ^4^

Pretomanid (PA‐824) is a new drug that was recently approved by the US Food and Drug Administration and by the European Medicines Agency for treating adult patients with extensively drug‐resistant or treatment‐intolerant or nonresponsive multidrug‐resistant pulmonary TB, in combination with bedaquiline and linezolid (BPaL regimen). Pretomanid is administered orally 200 mg once daily for 26 weeks; the BPaL regimen is taken with food. This agent is a nitroimidazooxazine compound active against drug‐sensitive and drug‐resistant MTB that has been shown to inhibit both protein and lipid synthesis in the bacterium and interfere with cell wall biosynthesis.^5^, ^6^, ^7^

Studies evaluating a potential proarrhythmic effect are required by regulatory agencies in the United States and Europe as part of the clinical development of drugs for noncardiac indications. Moxifloxacin is known to cause a mild increase in the QTc interval, and for this reason it is used as a positive control and assay sensitivity marker in thorough QT studies.^8^, ^9^ The present study was the first specifically aimed at thoroughly assessing the effect of pretomanid on cardiac repolarization in healthy subjects. In addition, because pretomanid 200 mg and moxifloxacin 400 mg are being evaluated together in an investigational regimen to treat pulmonary TB, it was important to evaluate whether there is increased risk of QT prolongation when the 2 drugs are also given together.

Cardiovascular and electrocardiographic (ECG) effects of pretomanid were evaluated in vitro and in preclinical and several clinical studies. In vitro, pretomanid is a weak inhibitor (IC_50_ of approximately 17 to 20 μM, or 6.1 to 7.2 μg/mL in a protein‐free medium) of the human ether‐à‐go‐go‐related gene channel, which plays a central role in cardiac repolarization. Drug‐induced prolongation of this phase of the cardiac cycle, as measured by the QT interval, is associated with torsade de pointes, which can lead to ventricular fibrillation and sudden cardiac death. Other in vitro cardiovascular assessments were conducted on the slow component of the delayed rectifier potassium current (IKs, KCNQ1/minK), as well as the calcium and late and peak sodium currents. Pretomanid showed inhibition of KCNQ1/minK and peak sodium currents with an IC_50_ of 23 and 29 μM (8.3 and 10.4 μg/mL), respectively, in protein‐free media. Pretomanid did not inhibit calcium or late sodium currents when tested up to 30 μM (10.8 μg/mL). According to a population pharmacokinetic (PopPK) model, the median steady‐state C_max_ is 3.2 μg/mL (C_max,free_, 0.44 μg/mL) for the recommended pretomanid clinical dose of 200 mg qd in the fed state.^10^

Across studies in conscious male cynomolgus monkeys, pretomanid resulted in either no QTc prolongation or marginal QTc prolongation. The no‐effect dose in the 15‐day monkey study in which some prolongation was observed was 150 mg/kg/day, which produced a C_max_ of 15.7 μg/mL. Clinical evidence suggests that there is minimal risk of QT prolongation when pretomanid is coadministered with moxifloxacin at exposures in the concentration range being explored in human clinical trials.^11^, ^12^, ^13^, ^14^

Unpublished nonclinical and clinical mass balance studies have shown that pretomanid is metabolized via a combination of reductive and oxidative metabolism. In vitro, cytochrome P450 (CYP) 3A4 is responsible for up to approximately 20% of pretomanid's metabolism. Pretomanid is not a substrate of CYP2C9, CYP2C19, or CYP2D6. Fourteen circulating metabolites of pretomanid have been quantified. Only 1 metabolite, trifluoromethoxybenzoic acid, was present at more than 10% (35%) of parent exposures. Important drivers of clinical pharmacokinetic variability are dosing with or without food and coadministration of medications that induce CYP3A4. Administering pretomanid with a high‐calorie, high‐fat meal increased exposure relative to the fasted state in a dose‐dependent manner. With a single dose of 200 mg, the geometric mean ratio fed/fasted of AUC_0‐∞_ was 188%.^15^ In the presence of rifampicin, efavirenz, and lopinavir/ritonavir, steady‐state AUC_0‐24_ of pretomanid administered under fasted conditions was reduced by 66%, 35%, and 17%, respectively.^16^ Population pharmacokinetic modeling found that the apparent clearance and volume of distribution of pretomanid scaled allometrically with weight and that clearance was 11% and 18% less in females than in males.^10^, ^17^

Pretomanid has limited potential for effects as a perpetrator. Unpublished nonclinical studies have found pretomanid not to induce CYP3A4, CYP2C9, or CYP2E1 and not to inhibit CYP1A2, CYP2C8, CYP2C9, CYP2C19, CYP2D6, or CYP3A4/5. Its lack of clinically relevant effects on CYP3A4 was confirmed in a clinical study with midazolam.^18^ Nonclinical studies have found pretomanid to have no clinically relevant effect on the transporters breast cancer resistance protein (BCRP), bile salt export pump (BSEP), multidrug and toxin extrusion 1 (MATE1), MATE2, organic anion transporter 1 (OAT1), OAT1B1, organic anion transporting polypeptide 1B3 (OATP1B3), organic cation transporter 1 (OCT1), OCT2, or p‐glycoprotein (P‐gp). However, it was found to inhibit OAT3, with a Ki of 2.22 μM (0.79 μg/mL). Also, it has been found to increase serum creatinine levels by inhibiting renal tubular creatinine secretion.^19^

## Material and Methods

### Study Design

This was a phase 1 single‐center, double‐blind, randomized, placebo‐controlled, 5‐period crossover study. The study was conducted in accordance with the Declaration of Helsinki, the International Conference on Harmonization Good Clinical Practice guidelines, and good clinical practices as required by 21 Code of Federal Regulations, at Quintiles Phase I Services (Overland Park, Kansas). All study‐related material was approved by Western Institutional Review Board (Olympia, Washington). Written informed consent was obtained from all study subjects before enrollment. The trial was registered with ClinicalTrials.gov, number NCT01674218.

This study had 4 objectives. First was to evaluate the effect of single‐dose administration of pretomanid 400 and 1000 mg versus placebo on the change from baseline of the individually corrected QT interval (QTcI). Second was to evaluate the effect of pretomanid with or without moxifloxacin on QT with Fridericia's correction (QTcF), QT with Bazett's correction (QTcB), and QT with a population correction derived from the study's data (QTcN), as well as non‐QT interval ECG parameters (heart rate [HR] and PR, QRS, and RR intervals). The third objective was to evaluate the pharmacokinetics (PK) of pretomanid and pretomanid plus moxifloxacin as well as the plasma concentration–QTc interval effect relationship of pretomanid and of pretomanid plus moxifloxacin. And finally, the fourth objective was to evaluate the safety and tolerability of a single dose of pretomanid with or without moxifloxacin.

Assay sensitivity was assessed using moxifloxacin as a positive control.

### Subjects

Healthy men and women aged between 18 and 45 years (inclusive) with a body mass index of 18 to 30 kg/m^2^ could be included if they met standard entry criteria for healthy subjects defined in the protocol. Subjects were excluded if any of the following were present: baseline QTcF interval > 440 milliseconds (men) or > 450 milliseconds (women) or a history of prolonged QTc interval or a family history of long QT syndrome or premature cardiac death or sudden death; clinically significant ECG abnormality; or ECGs with T‐wave morphology unfavorable for consistently accurate QT measurement. A total of 75 subjects were to be randomized to have at least 60 evaluable subjects.

### Sample Size

Under the assumption of 8‐millisecond residual variability within subjects, a 1‐sided significance level of 0.05, and time‐matched, placebo‐corrected QTc profile of (1, 1, 1, 2, 2, 3, 3, 5, 5, 4, 4, 3, 2, 1, 1) milliseconds, 60 completing subjects would yield >85% power to conclude that the QTc‐prolonging effect of PA‐824 400 mg administered alone is less than 10 milliseconds for all ECG assessments.^20^ To allow for a 20% withdrawal rate, 75 subjects were to be enrolled.

### Treatments

Study participants were randomized into 1 of 10 treatment sequences using a Williams Latin square design. Five study treatments were administered to each subject: treatment A, pretomanid placebo plus moxifloxacin placebo (negative control, referred to as placebo below); treatment B, pretomanid 400 mg plus moxifloxacin placebo (test, referred to as Pa400 below); treatment C, pretomanid 1000 mg plus moxifloxacin placebo (test, referred to as Pa1000 below); treatment D, pretomanid placebo plus moxifloxacin 400 mg (positive control, referred to as moxifloxacin below); and treatment E, pretomanid 400 mg plus moxifloxacin 400 mg (test, referred to as Pa400M below).

Treatments were administered by mouth on an empty stomach after an overnight fast. A light lunch was served following the assessments 4 hours postdose. Each treatment was given once in each of 5 treatment periods, followed by a washout period of at least 7 days (72 hours as inpatient in the clinical trial unit after dosing and at least 4 days as outpatient). Both subjects and clinical site investigators, as well as personnel at the National Institute of Allergy and Infectious Diseases, National Institutes of Health, TB Alliance, DynPort Vaccine Company, and Quintiles, were blinded to the treatments the subjects received.

### Dose Justification

Based on the sponsor's knowledge at the time the study was designed, the pretomanid 400‐mg single dose was intended to achieve the steady‐state exposure achieved in TB patients for the anticipated therapeutic dose of 200 mg once daily, and the pretomanid 1000‐mg dose was intended to serve as a supratherapeutic dose. Moxifloxacin 400 mg is the recommended dose approved for use in the clinic and is commonly used as a positive control in thorough QT/QTc studies to ensure assay sensitivity for detecting potentially clinically significant changes in the QT/QTc interval.^8^, ^9^

### Pharmacokinetics

Blood samples for the measurement of plasma concentrations of pretomanid were collected on the first day of each treatment period (study days 1, 8, 15, 22, and 29) at 0 hours (predose baseline) and 0.25, 1, 2, 3, 4, 5, 6, 7, 8, 10, and 12 hours postdose; 24 hours on days 2, 9, 16, 23, and 30 postdose; 48 hours on days 3, 10, 17, 24, and 31 postdose; 72 hours on days 4, 11, 18, 25, and 32 postdose; and 96 hours on days 5, 12, 19, 26, and 33 postdose.

The pretomanid plasma concentrations were measured using a validated ultraperformance liquid chromatography‐tandem mass spectrometric method (UPLC‐MS/MS) with an assay range of 10.0 to 10 000 ng/mL using 25.0 μL of plasma. A deuterated pretomanid (C_14_H_7_ **D_5_**F_3_N_3_O_5_) was used as internal standard. The high‐pressure liquid chromatography column was a Waters UPLC HSS C18, SB (P.N. 186004118). Samples were extracted using a Tomtec Quadra^®^ automated sample handling system. The mobile phase was 50:50:0.1 (CAN:H_2_O:HCOOH). MS/MS detection settings were multiple reaction monitoring positive mode with pretomanid transition *m/z* 359.9→174 and internal standard *m/z* 364.9→175. Freeze/thaw stability was 5 cycles at –20°C. PK data were analyzed by blinded personnel at Quintiles.

In the study protocol, it was defined that blood samples would not be assayed for moxifloxacin unless there were spurious ECG data on subjects treated with moxifloxacin, which would raise concern about the absorption of the drug. Analysis of Holter ECG data did not show any such concerns, and therefore, this analysis was never done.

### Pharmacodynamics

Twenty‐four‐hour ECGs were digitally obtained using a continuous Holter ECG recorder on day –1 and on the first day of each treatment period (days 1, 8, 15, 22, and 29). Data from day –1 were used in the formulas for QTcI and QTcN. Holter ECGs were collected 90, 60, and 30 minutes prior to dosing on the first day of each treatment period, and these 3 values were averaged for the determination of baseline in the calculations of ΔQTc. Individual digital ECGs were extracted from the continuous recording and analyzed predose and postdose at 0.25, 1, 2, 3, 4, 5, 6, 7, 8, 10, 12, and 24 hours. The replicate ECG intervals RR, PR, QRS, and QT plus ECG morphological abnormalities and interpretations were extracted from the digital ECG data for derivation of analysis variables, analysis, and reporting. Pharmacodynamic (PD) data were analyzed by blinded personnel at Quintiles.

### Safety

Safety was evaluated by a review of adverse events (AEs), clinical laboratory evaluations, vital signs, standard 12‐lead ECG results, physical examinations, and standard ophthalmology examinations (including visual acuity, pupil examination, confrontation fields, motility, and fundoscopy without mydriasis) from the time of first dose to the end of the clinical study. Severity of AEs was graded using the Division of Microbiology and Infectious Diseases toxicity assessment tables for studies with healthy subjects. Safety oversight and all data reviews were performed by blinded personnel prior to statistical analysis. A safety monitoring committee was also appointed to oversee this study.

### Statistical Analyses

#### Pharmacokinetics

Pharmacokinetic parameters were derived using noncompartmental methods with WinNonlin Professional version 5.2 (Pharsight Corp., Mountain View, California). The following pretomanid PK parameters were estimated by noncompartmental methods using actual elapsed time from dosing: C_max_, t_max_, AUC_0‐t_, AUC_0‐∞_, t_1/2_, and CL/F.

PK parameters were summarized by treatment using descriptive statistics. The dose proportionality between the pretomanid 400‐ and 1000‐mg doses and the effect of the coadministration of moxifloxacin on the PK of pretomanid 400 mg were investigated using a linear mixed model.

#### Pharmacodynamics

In the PD analysis, estimates of the individual (QTcI) and population (QTcN) correction factors were derived from a random coefficients linear mixed model for logQT on logRR using data from day –1 and predose values from all treatment periods.

The variables included the change from baseline of QTcI (ΔQTcI) and the changes from baseline of QT corrected by the other formulas (ΔQTcF, ΔQTcB, ΔQTcN). Other end points evaluated included HR (bpm); PR, QRS, and RR intervals (milliseconds); and ECG cardiac abnormalities (morphologies, rhythm, and conduction).

Analyses were conducted to evaluate the effects of pretomanid 400 and 1000 mg on cardiac repolarization as measured by ΔQTcI relative to placebo using a linear mixed model. At each point, the mean difference of ΔQTcI between a nonplacebo treatment and placebo, ΔΔQTcI, was estimated from the respective least‐squares means with the corresponding 90% 2‐sided confidence interval (CI) reported. No significant increase compared with placebo was concluded if the upper 90% confidence bound was below 10 milliseconds across all times under intersection‐union testing.

Statistical comparisons of the QTc end points (ΔQTcN, ΔQTcF, and ΔQTcB) plus ΔQT were also conducted for Pa400 and Pa1000 and Pa400M using the same analysis method described above for ΔQTcI.

Non‐QTc intervals (HR and PR, QRS, and RR intervals) were compared using the same statistical model as the primary analysis. However, no specific decision criteria were applied to the point estimates or CIs.

The statistical model for the ΔQTcI analysis was also used for assay sensitivity evaluation. A significant increase compared with placebo was concluded if the lower 90% confidence bound of ΔΔQTcI, averaged across the 1‐ to 4‐hour assessments after administration of moxifloxacin 400 mg was above 5 milliseconds. This analysis was repeated for the remaining QT/QTc end points.

The morphological waveform analysis of the ECG data from the Core ECG Laboratory at each time was used for the summary of new‐onset cardiac abnormalities (count and percent of subjects) for each treatment. New onset was determined programmatically and was defined as present on treatment, but not present during the baseline pretreatment day –1 of period 1 (including day 1 predose).

#### Pharmacokinetic‐Pharmacodynamic Relationship

A basic assessment of the relationship between PA‐824 plasma concentrations and the QTcI interval measured at the same protocol time was carried out. Initially, random coefficients linear mixed‐effects models were fitted to placebo‐corrected change‐from‐baseline QTcI intervals for only the PA‐824 400‐ and 1000‐mg treatment arms (treatment B and treatment C). The linear mixed model contained fixed effects for concentration, plus random coefficients per subject for concentration slopes and intercepts. The PA‐824 400 mg plus moxifloxacin arm (treatment E) was not included in modeling. Estimates of the mean ΔΔQTcI at the geometric mean PA‐824 C_max_ for the PA‐824 400 and 1000‐mg doses were made from the models with corresponding 2‐sided 90%CIs. Estimation at geometric mean C_max_ was intended to capture the population average maximum effect directly attributable to the expected maximum concentration for the population. Nonlinearity in concentration effects were assessed and modeled if assumptions of linear effects were inadequate.

Graphics were prepared with SAS version 9.2.

#### Safety

Safety assessments were summarized with descriptive statistics or frequency counts of treatment‐emergent AEs (TEAEs) for each treatment.

## Results

### Subject Characteristics and Disposition

In total, 194 subjects were screened, and 74 were randomized (Table 1). All 74 subjects received at least 1 dose of study drug; 69 subjects (93.2%) received all planned doses of study drug. Five subjects (6.8%) were prematurely withdrawn from the study: 4 because of TEAEs and 1 with a positive drug screen. Analyses of safety, PK, and PD were performed on all 74 subjects.

**Table 1:** Subject Demographics and Baseline Characteristics

Variable/Category	Subjects (n = 74)
Sex, n (%)	
Female	30 (40.5)
Male	44 (59.5)
Race, n (%)	
White	40 (54.1)
Black or African American	32 (43.2)
Native Hawaiian or Other Pacific Islander	1 (1.4)
American Indian or Alaska Native	1 (1.4)
Ethnicity, n (%)	
Hispanic or Latino	2 (2.7)
Not Hispanic or Latino	72 (97.3)
Age (y)	
Mean	30
SD	7
Range	18‐45
Screening height (cm)	
Mean	171.0
SD	9.2
Range	148.9‐185.1
Screening weight (kg)	
Mean	75.1
SD	12.5
Range	50.9‐102.1
BMI (kg/m^2^)	
Mean	25.6
SD	3.1
Range	18.4‐30.0

BMI, body mass index; SD, standard deviation.

#### Safety and Tolerability

No deaths or serious AEs occurred during the study. Four subjects were withdrawn from the study because of nonserious TEAEs: papular rash (mild, ie, grade 1, and related to Pa400M, as assessed by the investigator), ventricular extrasystoles (mild and related to placebo per investigator), sinus tachycardia (moderate, ie, grade 2, and not related to Pa400M, per investigator), and prolonged QTcF in 12‐lead ECG (mild and not related to placebo, per investigator).

The most frequently reported TEAEs overall (4 or more subjects [5.4%]) were contact dermatitis (31 subjects [41.9%]), decreased hemoglobin (30 subjects [40.5%]), headache (17 subjects [23.0%]), nausea (8 subjects [10.8%]), ECG QT prolonged (6 subjects [8.1%]), increased alanine aminotransferase (6 subjects [8.1%]), dizziness (5 subjects [6.8%]), increased aspartate aminotransferase (4 subjects [5.4%]), protein in urine (4 subjects [5.4%]), and ecchymosis (4 subjects [5.4%]); see Table 2. The most frequently reported study drug‐related TEAEs (4 or more subjects [5.4%]) were nausea (8 subjects [10.8%]), headache (6 subjects [8.1%]), ECG QT prolonged (4 subjects [5.4%]), and dizziness (4 subjects [5.4%]).

**Table 2:** Most Frequent (2 or More Subjects Overall) Treatment‐Emergent Adverse Events

SOC/Preferred Term^a^, ^b^	Trt A (n = 73)	Trt B (n = 74)	Trt C (n = 71)	Trt D (n = 71)	Trt E (n = 74)	Overall (n = 74)
Number of subjects with TEAEs, n (%)	29 (39.7)	31 (41.9)	28 (39.4)	35 (49.3)	27 (36.5)	61 (82.4)
Gastrointestinal disorders, n (%)	0	0	1 (1.4)	6 (8.5)	6 (8.1)	11 (14.9)
Nausea	0	0	1 (1.4)	5 (7.0)	3 (4.1)	8 (10.8)
General disorders and administration‐site conditions, n (%)	3 (4.1)	4 (5.4)	0	3 (4.2)	0	10 (13.5)
Fatigue	1 (1.4)	1 (1.4)	0	0	0	2 (2.7)
Irritability	0	0	0	2 (2.8)	0	2 (2.7)
Injury, poisoning, and procedural complications, n (%)	3 (4.1)	1 (1.4)	0	0	1 (1.4)	4 (5.4)
Laceration	1 (1.4)	1 (1.4)	0	0	0	2 (2.7)
Investigations, n (%)	14 (19.2)	13 (17.6)	17 (23.9)	16 (22.5)	14 (18.9)	47 (63.5)
Hemoglobin decreased	6 (8.2)	7 (9.5)	9 (12.7)	9 (12.7)	7 (9.5)	30 (40.5)
ALT increased	1 (1.4)	2 (2.7)	4 (5.6)	1 (1.4)	1 (1.4)	6 (8.1)
ECG QT prolonged	3 (4.1)	2 (2.7)	1 (1.4)	3 (4.2)	2 (2.7)	6 (8.1)
AST increased	1 (1.4)	0	1 (1.4)	2 (2.8)	0	4 (5.4)
Protein urine	1 (1.4)	1 (1.4)	0	1 (1.4)	1 (1.4)	4 (5.4)
Systolic blood pressure decreased	1 (1.4)	0	2 (2.8)	1 (1.4)	1 (1.4)	3 (4.1)
Red blood cells in urine	1 (1.4)	1 (1.4)	0	1 (1.4)	0	3 (4.1)
Blood CPK increased	0	0	1 (1.4)	1 (1.4)	0	2 (2.7)
Nervous system disorders, n (%)	2 (2.7)	7 (9.5)	4 (5.6)	8 (11.3)	8 (10.8)	21 (28.4)
Headache	2 (2.7)	5 (6.8)	4 (5.6)	5 (7.0)	7 (9.5)	17 (23.0)
Dizziness	0	1 (1.4)	0	3 (4.2)	1 (1.4)	5 (6.8)
Respiratory, thoracic, and mediastinal disorders, n (%)	1 (1.4)	1 (1.4)	1 (1.4)	1 (1.4)	0	4 (5.4)
Nasal congestion	1 (1.4)	0	1 (1.4)	1 (1.4)	0	3 (4.1)
Skin and subcutaneous tissue disorders, n (%)	13 (17.0)	9 (12.2)	11 (15.5)	13 (18.3)	8 (10.8)	34 (45.9)
Dermatitis contact	10 (13.7)	8 (10.8)	10 (14.1)	10 (14.1)	7 (9.5)	31 (41.9)
Ecchymosis	2 (2.7)	0	0	2 (2.8)	0	4 (5.4)

ALT, alanine aminotransferase; AST, aspartate aminotransferase; CPK, creatine phosphokinase; ECG, electrocardiogram; SOC, system organ class; TEAE, treatment‐emergent adverse event; Trt, treatment (A: placebo; B: Pa400; C: Pa1000; D:moxifloxacin; E: Pa400M).

^a^ The SOC subject totals can be higher than the preferred term subject totals because the SOC total can contain TEAEs that were experienced by only 1 subject, whereas the TEAEs listed by preferred term occurred in 2 or more subjects overall. If a subject experienced more than 1 episode of a TEAE, the event was counted only once within a preferred term. If a subject experienced more than 1 TEAE within an SOC, the subject was counted once for each preferred term and once for the SOC. The number of subjects experiencing a given TEAE across the treatment groups can be different than the overall total for that TEAE because a subject is counted only once in the overall total.

^b^ System organ class and preferred terms are from the Medical Dictionary for Regulatory Activities, version 15.1.

No meaningful differences in the frequency of TEAEs were noted among the treatments with the exception of nausea, headache, and dizziness. Nausea was reported by 5 subjects (7.0%) and 3 subjects (4.1%) who received moxifloxacin alone and Pa400M, respectively, compared with 1 subject (1.4%) who received Pa1000 and no subjects who received placebo or Pa400. Headache was reported by 5 subjects (6.8%) who received Pa400, 4 subjects (5.6%) who received Pa1000, 5 subjects (7.0%) who received moxifloxacin, and 7 subjects (9.5%) who received Pa400M compared with 2 subjects (2.7%) who received the placebo. Dizziness was reported by 3 subjects (4.2%) who received moxifloxacin compared with 1 subject (1.4%) each who received Pa400 and Pa400M and no subjects who received placebo or Pa1000. Of note, contact dermatitis was observed across all treatment arms with a clear link to ECG patch irritation.

One subject who received Pa1000 experienced a grade 3 TEAE of increased blood creatine phosphokinase (2087 U/L; upper limit of normal, 294 U/L), which was considered related to increased physical activity rather than study drug per the investigator. Apart from 8 subjects with moderate TEAEs, all other events were mild in severity.

QTcF findings from safety standard 12‐lead ECGs were consistent with the digital Holter ECG data obtained for the PD objectives of the study. The mean QTcF values observed were well below the grade 1 protocol‐specified limits of 441 milliseconds (male) and 451 milliseconds (female). Mean change‐from‐baseline QTcF was somewhat higher for the 2 moxifloxacin‐containing treatments (moxifloxacin, Pa400M) compared with the other 3 moxifloxacin‐free treatments (placebo, Pa400, Pa1000). At the 2‐hour postdose assessment, mean QTcF change from baseline was 8 and 9 milliseconds for moxifloxacin and Pa400M, respectively. For placebo, Pa400, and Pa1000, 2‐hour postdose QTcF change from baseline was –1, –2, and 0 milliseconds, respectively. This was also consistent with the digital ECG QTcF data obtained for the PD objectives of the study.

Clinical laboratory, vital sign, physical examination, Snellen visual acuity, and ophthalmological data did not reveal any clinically meaningful trends or changes throughout the study. In addition, no meaningful differences were observed between the treatments. Decreases over time in red blood cell count, hematocrit, and hemoglobin were observed in all treatment groups, which were likely related to blood loss from blood collection procedures.

One pregnancy was detected at the final follow‐up study visit 12 days after the last treatment in a subject with previously negative pretreatment urine pregnancy tests in all periods. Follow‐up information on the pregnancy was collected. The last available record obtained approximately 1 month before the due date indicated that the pregnancy was proceeding normally. No further information regarding the pregnancy or its outcome was available.

### Pharmacokinetics

**Figure 1:**
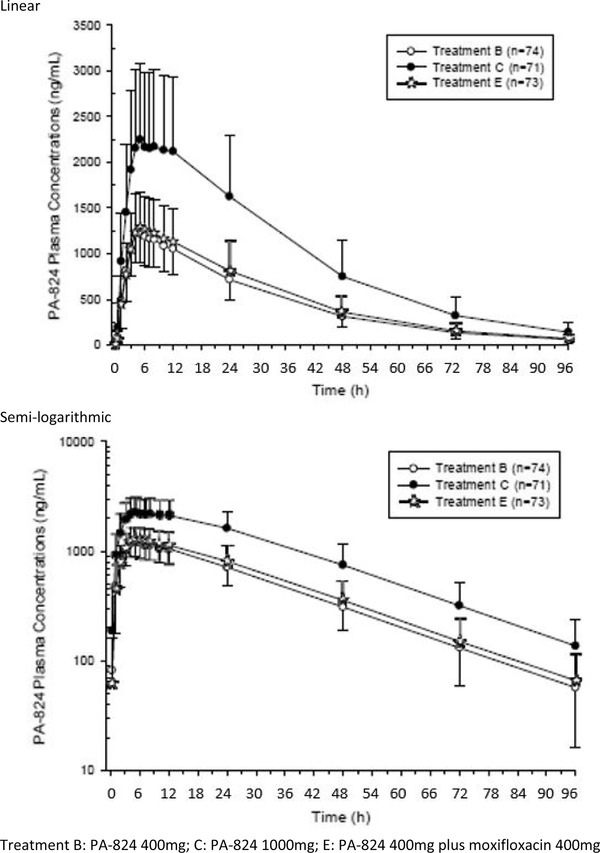
Mean (SD) PA‐824 plasma concentration‐time profiles by treatment on linear and semi‐logarithmic scales. Treatment B: PA‐824 400 mg; treatment C: PA‐824 1000 mg; treatment E: PA‐824 400 mg plus moxifloxacin 400 mg.

Pretomanid PK parameters (arithmetic mean ± SD) are summarized by treatment in Table 3. Arithmetic mean ± SD plasma concentrations of pretomanid at corresponding times, using both linear and logarithmic concentration axes, are presented in Figure 1.

**Table 3:** Arithmetic Mean (SD) Pretomanid (PA‐824) Plasma Pharmacokinetic Parameters by Treatment

Treatment	AUC_0‐∞_ (μ·h/mL)	AUC_0‐t_ (μg·h/mL)	C_max_ (μg/mL)	T_max_^a^ (h)	t_1/2_ (h)	CL/F (L/h)
B: PA‐824 400 mg (Pa400), n = 74	43.0 (13.1)	41.2 (12.1)	1.31 (0.347)	4.63 (1.08‐12.08)	19.1 (4.15)	10.1 (2.77)
C: PA‐824 1000 mg (Pa1000), n = 71	93.1 (38.8)	89.0 (36.2)	2.48 (0.980)	5.08 (1.10‐24.12)	19.1 (3.74)	12.2 (3.99)
E: PA‐824 400 mg plus moxifloxacin 400 mg (Pa400M), n = 73	47.3 (18.2)	45.3 (16.8)	1.37 (0.442)	5.08 (1.08‐24.08)	18.8 (3.84)	9.54 (3.22)

AUC, area under the concentration‐time curve; C_max_, maximum concentration; T_max_, time to maximum concentration; t_1/2_, half life.

^a^Median and range presented for T_max_.

Median time to maximum concentration (t_max_) was similar for both pretomanid doses and occurred approximately 4.5 to 5 hours postdose. Mean values of C_max_ were 1.31, 2.48, and 1.37 μg/mL for a pretomanid dose of 400, 1000, and 400 mg coadministered with moxifloxacin.

The geometric mean maximum concentration (C_max_) was 1.3 and 2.3 μg/mL for Pa400 and Pa1000, respectively. The geometric mean total area under the concentration‐time curve, that is, time 0 extrapolated to infinite time, AUC_0‐∞_, was 41.2 and 86.8 μg·h/mL, for Pa400 and Pa1000, respectively. A 2.5‐fold increase in the pretomanid dose from 400 to 1000 mg resulted in a 2.2‐fold increase in mean AUC and a 1.9‐fold increase in C_max_. The increase in AUC met the protocol‐defined criterion for dose proportionality (90%CIs for LS mean ratios of treatment C compared with treatment B were within the limits of 200% to 312.5%), but the increase in C_max_ was judged to be less than proportional to the increase in dose.

The PK of pretomanid was not affected by the coadministration of moxifloxacin. The 90%CIs for the AUC and C_max_ least‐squares mean ratios comparing Pa400M with Pa400 alone were contained within the limits of 80% to 125%.

A subset of subjects had quantifiable predose levels of pretomanid in a treatment period when the previous treatment period included pretomanid (see below). In no instance did the predose concentration exceed a value ≥ 5% of the subject's C_max_, and these treatment periods were included in all PK analyses.

### Pharmacodynamics

As a result of the quantifiable predose pretomanid concentrations observed in the PK analysis, a decision was made to analyze the predose samples collected during placebo and moxifloxacin treatment periods. Treatment periods in which pretomanid was present at baseline despite the washout were excluded from the key PD analysis because of the potential impact on the ECG at the initial extraction times including the baseline observation. This affected 12 subjects with placebo, 9 with Pa400, 5 with Pa1000, 14 with moxifloxacin, and 7 with Pa400M. A sensitivity analysis using the same methodology included all treatment periods with quantifiable pretomanid concentrations at baseline to assess the impact of their exclusion (see below).

#### Assay Sensitivity

**Figure 2:**
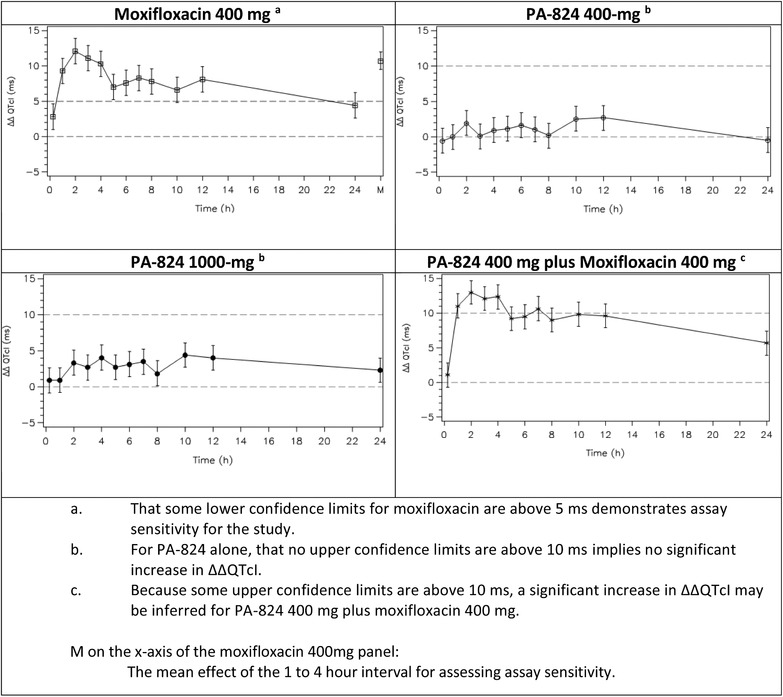
Least‐squares mean differences in QTcI and 90% confidence intervals between study drugs.

The least‐squares mean ΔΔQTcI value for moxifloxacin alone averaged across the 1‐ to 4‐hour postdose time frame was 10.7 milliseconds. The lower limit of the 90%CI was 9.5 milliseconds, which exceeded the 5‐millisecond threshold and thus established assay sensitivity for a thorough QTc study (Figure 2).

#### QTcI

The maximum least‐squares mean ΔΔQTcI value for the 400‐mg dose of pretomanid administered alone was 2.7 milliseconds and for the 1000‐mg dose of pretomanid was 4.4 milliseconds. The upper limits of the 90%CIs did not exceed 4.4 milliseconds for the 400‐mg dose or 6.1 milliseconds for the 1000‐mg dose; thus, both were below 10 milliseconds. The least‐squares mean ΔΔQTcI values and associated 90%CIs following the administration of Pa400M exceeded 10 milliseconds at multiple times during the observation period. This was similar to the effect of moxifloxacin administered alone (see Figure 2).

#### Other QTc Variables

Least‐squares mean ΔΔQTcN, ΔΔQTcF, and ΔΔQTcB values for moxifloxacin alone averaged across the 1‐ to 4‐hour postdose time frame were ≥10.0 milliseconds. The lower limits of the 90%CIs associated with these least‐squares mean values were ≥8.8 milliseconds. This established assay sensitivity for all 3 non‐QTcI QTc intervals.

The maximum least‐squares mean values for ΔΔQTcN, ΔΔQTcF, and ΔΔQTcB for the 400‐mg dose of pretomanid were 2.7, 2.3, and 3.8 milliseconds, respectively, and the upper limits of the 90%CIs did not exceed 6.0 milliseconds. The maximum least‐squares mean values for the 1000‐mg dose of pretomanid were 4.5, 4.6, and 5.0 milliseconds, and the upper limits of the 90%CIs did not exceed 7.2 milliseconds. Thus, the upper confidence limits were all below the 10‐millisecond threshold.

The least‐squares mean ΔΔQTcN, ΔΔQTcF, and ΔΔQTcB values and associated 90%CIs following the administration of Pa400M exceeded 10 milliseconds at multiple times during the observation period. This was similar to the effect of moxifloxacin administered alone.

#### Sensitivity Analysis

For ΔQTcI, a sensitivity analysis identical to the analysis described above was performed that included all treatment periods with quantifiable pretomanid concentrations at baseline. The results were very similar to the original analysis, demonstrating that the inclusion or exclusion of subjects with carryover pretomanid concentrations from the preceding treatment period had no impact on the study results.

#### Non‐QT ECG Variables

Based on the results excluding treatment periods with quantifiable predose pretomanid concentrations, pretomanid administered alone (Pa400 and Pa1000) had little effect on HR and the RR interval. Changes in HR and the RR interval were slightly larger in treatment groups containing moxifloxacin (moxifloxacin and Pa400M). The maximum least‐squares mean placebo‐corrected change‐from‐baseline values observed in HR for Pa400, Pa1000, moxifloxacin, and Pa400M were 1.8, 1.2, 3.5, and 4.4 bpm, respectively. There was also little effect on the PR or QRS interval observed in any treatment group. The largest least‐squares mean placebo‐corrected change‐from‐baseline values observed in the PR and QRS intervals occurred with Pa400M and were –4.1 and –0.9 milliseconds, respectively.

#### Categorical Analysis

No subject receiving pretomanid or moxifloxacin alone had an observed QTcI, QTcN, or QTcF value that exceeded 450 milliseconds or a change from baseline in QTcI, QTcN, and QTcF that exceeded 30 milliseconds. Two subjects receiving Pa400M had a change from baseline in QTcI between 30 and 60 milliseconds. No subject in any treatment group had an observed QTcB that exceeded 480 milliseconds or a change from baseline in QTcB that exceeded 60 milliseconds. One subject receiving moxifloxacin and 2 subjects receiving Pa400M had an observed QTcB value that was greater than 450 milliseconds but less than 480 milliseconds.

#### New‐Onset Morphological Changes

The number of subjects experiencing a new‐onset cardiac abnormality in the treatment groups containing pretomanid and/or moxifloxacin was comparable to the number of subjects in the placebo group. The percentage of subjects experiencing a new‐onset cardiac abnormality while receiving pretomanid and/or moxifloxacin ranged from 3.0% to 7.0%. By comparison, the percentage of subjects in the placebo group was 8.2%. The most commonly reported abnormalities were nonspecific T‐wave abnormality and abnormal flat T wave.

#### Pharmacokinetic‐Pharmacodynamic Relationships

**Figure 3:**
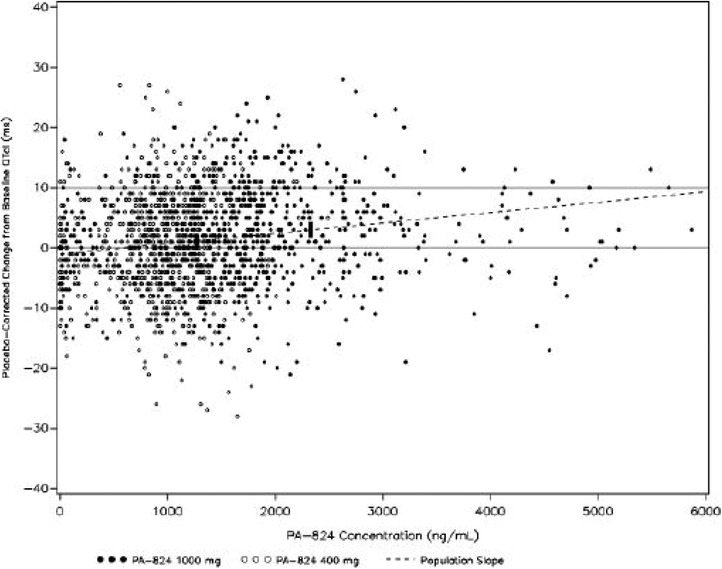
Individual ΔΔQTcI values versus PA‐824 concentrations on a linear scale (PA‐824 400‐ and 1000‐mg treatments combined). The solid line at 0 milliseconds is the no‐effect reference line. The solid line at 10 milliseconds is the threshold reference line. Population slope is estimated from random coefficient regression. Vertical bars present the 90%CI for placebo‐corrected change‐from‐baseline QTcI at the geometric mean C_max_ for each PA‐824 dose level.

Individual ΔΔQTcI values versus PA‐824 concentrations on a linear scale (PA‐824 400‐ and 1000‐mg treatments combined) are presented in Figure 3.

The slope of the linear relationship between ΔΔQTcI and pretomanid concentration was positive, with a magnitude of 1.72 ms/μg/mL (90%CI, 1.09–2.36 ms/μg/mL). Similarly, the slope of the linear relationship between ΔΔQTcF and pretomanid concentration was positive, with a magnitude of 1.72 ms/μg/mL (90%CI, 1.12–2.32 ms/μg/mL). Neither CI includes zero, indicating a significant relationship. However, as can be seen in Figure 2, there may have been a delayed response of QTc to pretomanid exposure, which was not accounted for in the model.

Model prediction of ΔΔQTcI at the geometric mean of C_max_ observed with the pretomanid 400‐mg dose was 1.17 milliseconds (90%CI, 0.23–2.12 milliseconds). Similarly, the model prediction of ΔΔQTcI at the geometric mean C_max_ observed with the pretomanid 1000‐mg dose was 3.00 milliseconds (90%CI, 1.72–4.29 milliseconds).

## Discussion

Drug‐induced prolongation of cardiac repolarization is a source of concern and a common cause of the restriction or withdrawal of already‐approved drugs. Therefore, this study was conducted to thoroughly assess the effect of pretomanid on QT/QTc interval prolongation. In addition, because a regimen including pretomanid 200 mg and moxifloxacin 400 mg was being evaluated for its use in the treatment of patients with pulmonary TB^11^ at the time this study was conducted, moxifloxacin was not only used as a positive control in this study, but was also studied in combination with pretomanid to evaluate whether there is an increased risk of QT prolongation when the 2 drugs are given together. Currently, a phase 2‐3 study is ongoing with the regimen of pretomanid‐moxifloxacin‐bedaquiline‐pyrazinamide.

The investigational products administered in this study were generally well tolerated when administered orally once in each of 5 treatment periods. There were no deaths or serious AEs during the study. Clinical chemistry, vital signs, physical examination, Snellen visual acuity, and ophthalmological examination data did not reveal any clinically meaningful trends or changes throughout the study. In addition, no meaningful differences were observed between active and placebo treatments in these assessments.

Pretomanid concentrations following single 400‐ or 1000‐mg doses were not associated with any QT interval prolongation of clinical concern. The maximum least‐squares mean ΔΔQTcI value for the 400‐mg dose of pretomanid was 2.7 milliseconds, and the upper limits of the 90%CIs did not exceed 4.4 milliseconds. The maximum least‐squares mean ΔΔQTcI value for the 1000‐mg dose of pretomanid was 4.4 milliseconds, and the upper limits of the 90%CIs did not exceed 6.1 milliseconds. The results of the analyses based on QTcN, QTcF, and QTcB were similar to the results of the analysis based on QTcI.

Moxifloxacin did not alter the PK of pretomanid, and the effect of pretomanid 400 mg plus moxifloxacin 400 mg on QTcI was consistent with the effect of moxifloxacin alone (see Figure 2).

Some transient elevations were observed in QTcN, QTcB, and QTcF, primarily during the active moxifloxacin treatments (treatments D and E); however, these events were not accompanied by any other clinically relevant cardiac abnormalities. Pretomanid had little effect on the non‐QT‐related ECG variables.

The average maximal exposures for the 2 pretomanid doses in this study were only approximately one‐half and two‐thirds of typical maximal exposures expected in clinical practice (3.2 μg/mL) when pretomanid 200 mg in the context of the BPaL regimen is administered with food. Thus, not only was supratherapeutic exposure not achieved in this study, but clinical maximal exposures were also underrepresented. This was a weakness of the study, mainly because at the time the study was initiated, it was not known that the clinical dose would require administration with food in the context of the approved regimen or of regimens in development.

Nonetheless, this study does add to the knowledge base about pretomanid's cardiac safety. According to the PopPK model,^10^ the average concentration over a dosing interval for the clinical regimen at steady state has a median value of 2.4 μg/mL. Thus, the average maximal exposure of Pa1000 in this study was approximately equal to the average exposure expected in clinical practice.

This study also adds value in association with the work of Li et al.^12^ They modeled pretomanid's exposure‐QTc relationship using data from phase 2‐3 studies, in some of which pretomanid was administered at its recommended clinical dose with food, yielding clinical maximal exposures, and in 1 of which pretomanid was administered fasted but at doses up to 1200 mg for 14 days, so that accumulation did yield supratherapeutic exposures. That work has an advantage of a more relevant exposure range but may be considered to have a weakness because of the source of the data being clinical studies, without full placebo profiles of ECGs and where exposure‐response analysis of QTc was a post hoc evaluation. The work reported here is based on data from a well‐controlled thorough QT/QTc study. To the extent that results from the 2 analyses agree, they complement and strengthen one another.

In Li et al,^12^ the estimated slope and 90%CI for the relationship between ΔΔQTcF and pretomanid concentration was 1.54 ms/μg/mL (1.21‐1.88 ms/μg/mL), whereas the corresponding value in this study was 1.72 ms/μg/mL (1.12‐2.32 ms/μg/mL). In the former case, the model for ΔΔQTcF did not include an intercept, so the predicted value of ΔΔQTcF at a pretomanid concentration of 3.2 μg/mL is 3.2 × 1.54 = 4.9 milliseconds. In the present work, an intercept was included and estimated to be –1.01, so the predicted value of ΔΔQTcF is –1.01 + 3.2 × 1.72 = 4.5 milliseconds. Thus, the 2 sets of results do agree and support a conclusion of at most a modest impact on QTc at clinical exposures. However, as noted previously, the modeling analysis in this study did not account for a possible delay in the QT response, and the same was true for the work of Li et al.^12^

The pharmacokinetics of pretomanid in subjects with severe hepatic or renal impairment (supratherapeutic exposures) have not yet been evaluated; therefore, whether and how much pretomanid concentration might change in such subjects and the consequences for QT interval prolongation are unknown.

Thus, even though supratherapeutic exposures of pretomanid were not achieved in this study, taken in context with other results, namely, the PK QT modeling work by Li et al,^12^ the findings here contribute to the favorable assessment of cardiac safety for pretomanid.

## Funding

The project described was supported by contract number HHSN272200800024C from the United States National Institute of Allergy and Infectious Diseases to DVC and by TB Alliance with support from Australia's Department of Foreign Affairs and Trade, the Bill & Melinda Gates Foundation, Germany's Federal Ministry of Education and Research through KfW, Irish Aid, Netherlands Ministry of Foreign Affairs, and the United Kingdom Department for International Development.

## Disclaimer

Blaire Osborn contributed to this article while employed in the Division of Microbiology and Infectious Diseases, National Institute of Allergy and Infectious Diseases, National Institutes of Health. This article reflects the views of the authors and should not be construed to represent the US Food and Drug Administration's views or policies.

## Author Contributions

All authors contributed to the writing and critically revised the manuscript, reviewed and approved the final version of the manuscript, and agreed to be accountable for all aspects of the work.
